# Cardiac safety of tiotropium in patients with cardiac events: a retrospective analysis of the UPLIFT® trial

**DOI:** 10.1186/s12931-015-0216-4

**Published:** 2015-06-02

**Authors:** Donald P Tashkin, Inge Leimer, Norbert Metzdorf, Marc Decramer

**Affiliations:** Department of Medicine, David Geffen School of Medicine at UCLA, Los Angeles, CA USA; Medical Affairs, Boehringer Ingelheim Pharma GmbH & Co KG, Ingelheim, Germany; Respiratory Division, University of Leuven, Leuven, Belgium

**Keywords:** UPLIFT®, Cardiac safety, Tiotropium HandiHaler®, COPD

## Abstract

**Background:**

Tiotropium is an anticholinergic bronchodilator for symptom relief and reducing exacerbations with an established safety profile in patients with chronic obstructive pulmonary disease (COPD). Using data from the 4-year Understanding Potential Long-term Impacts on Function with Tiotropium (UPLIFT®) study, we re-evaluated the safety of tiotropium HandiHaler® in patients who experienced recent myocardial infarction (MI), heart failure or unstable rhythm disorder during the study.

**Methods:**

A *post-hoc* analysis of all-cause mortality and serious cardiac adverse events (cardiac SAEs), including cardiac deaths and death unknown, was conducted in patients who had experienced cardiac arrhythmia, MI or cardiac failure during UPLIFT® and who completed the study. Descriptive analyses were performed.

**Results:**

Most patients experiencing cardiac events, for which they would have been excluded at baseline, remained in the trial. Kaplan-Meier analyses revealed a trend to later occurrence of cardiac SAEs with tiotropium HandiHaler® versus placebo. Patients who experienced a cardiac event and continued in UPLIFT® were not found to be at subsequently increased risk of all-cause mortality or cardiac SAEs with tiotropium treatment. Evaluation of deaths by major adverse cardiac events composite endpoints also showed that patients treated with tiotropium were not at increased risk of mortality or cardiac SAEs compared with placebo.

**Conclusions:**

Risk of cardiac events, mortality or SAEs was not increased by tiotropium in patients experiencing cardiac events for which they would have been excluded at study baseline. The findings support the cardiac safety of tiotropium HandiHaler® in patients with COPD.

**Electronic supplementary material:**

The online version of this article (doi:10.1186/s12931-015-0216-4) contains supplementary material, which is available to authorized users.

## Introduction

Tiotropium is a once-daily maintenance anticholinergic bronchodilator for the relief of symptoms and reducing exacerbations in patients with chronic obstructive pulmonary disease (COPD) [1-3]. Two formulations have been developed (SPIRIVA®, Boehringer Ingelheim, Ingelheim am Rhein, Germany): tiotropium HandiHaler® 18 μg once daily, a dry powder inhaler; and tiotropium Respimat® 5 μg (two puffs of 2.5 μg) once daily. Respimat® was developed to provide a more efficient drug delivery system, with increased deposition in the lung to allow for a reduced nominal dose versus Handihaler®, a delivered dose independent of inspiratory effort and ease of co-ordination of actuation and inhalation [4,5]. Pharmacokinetic analysis indicates that systemic exposure is similar for tiotropium 18 μg via HandiHaler® and tiotropium 5 μg via Respimat® [6,7]; clinical studies have also shown that the Tiotropium Respimat® is non-inferior to HandiHaler® for lung function outcomes and rescue medication use [6,7], as well as exacerbation risk [8].

A reduction in mortality versus placebo was observed in the Understanding Potential Long-term Impacts on Function with Tiotropium (UPLIFT®) study, a randomised, double-blind trial in which tiotropium HandiHaler® was compared with placebo in almost 6000 patients over a period of 4 years [9]. In this study, for the protocol-defined study period up to Day 1440, among patients for whom vital status information was available, 921 patients died: 14.4% in the tiotropium group, 16.3% in the placebo group (hazard ratio: 0.87; 95% confidence interval [CI]: 0.76–0.99) [9]. In line with the findings from UPLIFT®, other meta-analyses and data reviews have also failed to find any increases in all-cause mortality in patients with COPD [10-13].

However, safety concerns were raised when a *post-hoc* pooled analysis of three 1-year and one 6-month placebo-controlled trials found that tiotropium Respimat® 5 μg was associated with a higher number of fatal events than placebo, although the difference was not statistically significant. The difference was particularly found in patients with cardiac rhythm disorders at randomisation [14]. The contribution of a rhythm disorder to the fatal outcome was uncertain and a causal relationship with tiotropium Respimat® has not been established. Subsequent systematic reviews and meta-analyses of the same clinical dataset by different authors have also described an increase in mortality associated with Respimat® [15-17]. However, the TIOtropium Safety and Performance In Respimat® (TIOSPIR™) study found no difference in mortality with tiotropium Respimat® versus HandiHaler® with respect to the risk of death, and causes of death were similar between the groups [8].

The debate surrounding potential differential effects of HandiHaler® and Respimat® on mortality associated with tiotropium has led to discussion and re-evaluation of previous clinical trials in COPD to understand the validity of the findings. Most recently, the generalisability of the results of the 4-year UPLIFT® trial comparing tiotropium with placebo was called into question, based on potential eligibility of a hospitalised patient population with COPD in New Zealand for UPLIFT® [18]. In their evaluation of 100 patients who were prescribed tiotropium, the authors concluded that 38% (95% CI: 28.5–48.3) would have been ineligible for UPLIFT® at the time of hospital discharge due to recent cardiovascular (CV) co-morbidity or moderate to severe renal impairment. While the interpretation of the findings from this study were challenged by the authors of UPLIFT® [19], it was recognised that analysis of outcomes of patients experiencing cardiac events during the 4-year UPLIFT® study could add valuable data to the debate on the potential effects of tiotropium on CV and overall mortality. It has recently been noted that, in addition to looking at overall mortality or composite CV endpoints (such as major adverse CV events [MACE]), specific cardiac outcomes (such as myocardial infarction [MI]) should also be considered, to avoid masking a potential treatment effect on a particular type of event [20].

Patients experiencing specific recent cardiac events before the baseline of UPLIFT® were excluded from participation in the trial. Therefore, to evaluate tiotropium HandiHaler® safety in patients with recent cardiac events, we conducted *post-hoc* analyses of all-cause mortality and serious cardiac adverse events (cardiac SAEs), including cardiac deaths and death unknown, in patients who experienced cardiac arrhythmia, MI or cardiac failure during the conduct of the UPLIFT® study.

## Methods

Full details of the UPLIFT® methodology, including patient eligibility criteria, have been published previously [9].

### Study design and patients

UPLIFT® was a 4-year, randomised, double-blind, placebo-controlled, parallel-group trial involving patients with moderate to very severe COPD. All patients gave written informed consent. The study was approved by local ethical review boards in each center (see Additional file 1) and conducted in accordance with the Declaration of Helsinki.

Patients were excluded from UPLIFT® if they had a recent history (≤6 months) of MI, any unstable or life-threatening cardiac arrhythmia or cardiac arrhythmia requiring intervention or a change in drug therapy within the past year or hospitalisation for heart failure (New York Heart Association Class III or IV) within the past year. Patients were randomised to 18 μg of tiotropium or a matching placebo once daily, delivered through the HandiHaler® [9]. All respiratory medications, except other inhaled anticholinergic drugs, were permitted during the trial.

After randomisation to treatment groups, clinic visits occurred at 1 and 3 months, then every 3 months throughout the 4-year study period. Reports of adverse events (AEs) were collected at each visit. An independent data and safety monitoring committee reviewed data throughout the trial.

### Analysis of outcomes in patients experiencing a cardiac event during the study

Patients who were selected for the *post-hoc* analyses had to have experienced a cardiac event with onset during treatment, but following the first occurrence of the cardiac event, they did not withdraw from UPLIFT® (either due to the event or for another reason).

Three types of cardiac events were investigated: arrhythmia (defined as Standardised Medical Dictionary for Regulatory Activities [MedDRA] Query [SMQ] Cardiac arrhythmias sub-SMQ Cardiac arrhythmia terms), MI (defined as SMQ ischaemic heart disease sub-SMQ Myocardial Infarction [broad]) and cardiac failure (defined as SMQ Cardiac Failure [narrow]).

Outcomes in patients treated with tiotropium were compared with those receiving placebo. Evaluations included the time to onset of the initial cardiac AE and occurrence of SAEs, cardiac SAEs (using MedDRA version 16.0 definitions) and fatal AEs (FAEs) in the time after the initial cardiac AE.

For the vital status analysis, FAEs were counted if the death occurred ≥1 day following the first cardiac event of interest (arrhythmia, MI or cardiac failure) and within 1440 days of drug start. For the on-treatment analysis, events were counted if the event occurred ≥1 day following the first cardiac event of interest (arrhythmia, MI or cardiac failure) until cessation of treatment plus 30 days.

A composite endpoint of MACE was included in the analyses. This endpoint represents fatal events in the system organ class (SOC) cardiac disorders and SOC vascular disorders combined with MI (fatal and non-fatal), stroke (fatal and non-fatal) and the following preferred terms: sudden death, sudden cardiac death and cardiac death. For the composite endpoint of fatal MACE, non-fatal MI and non-fatal stroke were removed. As a sensitivity analysis, fatal MACE plus the preferred term ‘death unknown’ was also analysed.

### Statistical analysis

Descriptive statistical analysis only is presented.

## Results

### Cardiac and mortality outcomes for patients with cardiac arrhythmia during the UPLIFT® study

**Figure 1:**
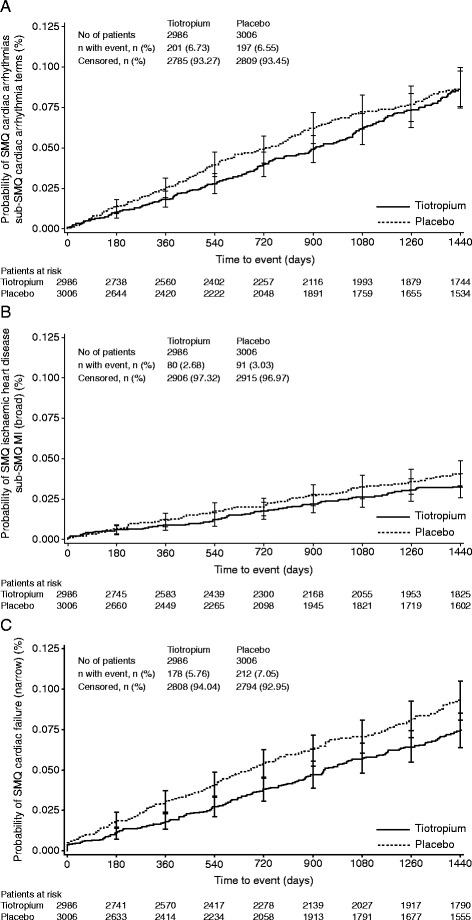
Kaplan-Meier analysis of time to onset of the first event. **(A)** Cardiac arrhythmia, **(B)** MI and **(C)** cardiac failure. Time to event is censored at Day 1440. MI, myocardial infarction, SMQ, Standardised Medical Dictionary for Regulatory Activities Query.

In the UPLIFT® trial, 5993 patients were randomly assigned, 2987 to receive tiotropium and 3006 to receive placebo. During the study, 400 patients experienced cardiac arrhythmia: 197 within the placebo arm, and 203 in the tiotropium treatment arm. Immediately following the first event, there were 26 discontinuations: 16 within the placebo arm, 10 in the tiotropium treatment arm. Kaplan-Meier analysis revealed a trend to later onset of the initial cardiac arrhythmia event in the tiotropium group (Figure 1A).

Mean treatment duration after the first cardiac arrhythmia event was 576.0 days (standard deviation [SD]: 454.3) for placebo and 518.8 days (SD: 438.0) for tiotropium patients. Among those patients who were administered placebo and who experienced a cardiac arrhythmia during the study, 60.2% (109/181) subsequently experienced an SAE and 20.4% (37/181) experienced a cardiac SAE. Similar percentages of patients experienced SAEs (53.9% [104/193]) and cardiac SAEs (22.8% [44/193]) in the tiotropium arm (Table 1).

**Table 1:** **SAEs (on-treatment), FAEs (on-treatment/vital status follow-up) and MACE (on-treatment + 30 days) in patients with cardiac events during UPLIFT** **®**

	**Patients with event, n (%)**					
**First event**	**Cardiac arrhythmia** ^*^		**MI** ^*^		**Cardiac failure** ^*^	
**Treatment arm/**	**Placebo**	**Tiotropium**	**Placebo**	**Tiotropium**	**Placebo**	**Tiotropium**
**Subsequent event**	**(n = 181)**	**HandiHaler** **®**	**(n = 68)**	**HandiHaler** **®**	**(n = 186)**	**HandiHaler** **®**
		**18 μg (n = 193)**		**18 μg (n = 63)**		**18 μg (n = 155)**
SAEs (total)	109 (60.2)	104 (53.9)	38 (55.9)	43 (68.3)	113 (60.8)	91 (58.7)
Cardiac	37 (20.4)	44 (22.8)	19 (27.9)	18 (28.6)	44 (23.7)	44 (28.4)
Vital status FAE (total)	38 (21.0)	34 (17.6)	11 (16.2)	8 (12.7)	41 (22.0)	34 (21.9)
Cardiac	12 (6.6)	8 (4.1)	3 (4.4)	3 (4.8)	15 (8.1)	9 (5.8)
General disorders^†^	6 (3.3)	6 (3.1)	1 (1.5)	1 (1.6)	6 (3.2)	3 (1.9)
On-treatment FAE (total)	36 (19.9)	28 (14.5)	11 (16.2)	6 (9.5)	31 (16.7)	30 (19.4)
Cardiac	13 (7.2)	9 (4.7)	3 (4.4)	3 (4.8)	11 (5.9)	9 (5.8)
General disorders^†^	4 (2.2)	3 (1.6)	1 (1.5)	0 (0.0)	3 (1.6)	2 (1.3)
Fatal MACE	15 (8.3)	10 (5.2)	5 (7.4)	3 (4.8)	13 (7.0)	11 (7.1)
Fatal MACE (including death unknown)	18 (9.9)	13 (6.7)	5 (7.4)	3 (4.8)	16 (8.6)	11 (7.1)
MACE	25 (13.8)	15 (7.8)	11 (16.2)	6 (9.5)	23 (12.4)	16 (10.3)

*First event did not lead to discontinuation. †SOC: general disorders and administration site conditions (death, sudden death, sudden cardiac death). FAE, fatal adverse event, MACE, major adverse cardiovascular event, MI, myocardial infarction, SAE, serious adverse event, SOC, system organ class; UPLIFT®, Understanding Potential Long-term Impacts on Function with Tiotropium.

For patients who experienced a cardiac arrhythmia, FAEs on-treatment affected 19.9% of patients receiving placebo and 14.5% of patients treated with tiotropium (Table 1). Vital status analysis was similar, with FAEs occurring slightly more frequently in those who had received placebo (21.0%) compared with tiotropium (17.6%) (Table 1).

Evaluation of events on-treatment by MACE endpoints did not suggest an increase in events with tiotropium compared with placebo for any of the measures (Table 1; Additional file 2 also provides a categorical breakdown of the incidence of fatal MACE, fatal MACE [including death unknown] and MACE). For patients with a cardiac arrhythmia, the incidence of on-treatment MACE was 13.8% in the placebo arm compared with 7.8% in the tiotropium arm. Fatal on-treatment MACE affected 8.3% of patients receiving placebo and 5.2% of patients treated with tiotropium; the incidence of fatal on-treatment MACE including death unknown was 9.9% and 6.7%, respectively.

### Cardiac and mortality outcomes for patients with an MI during the UPLIFT® study

During the study period, 172 patients experienced an MI: 92 within the placebo arm, 80 in the tiotropium arm. Following the first event, there were 41 discontinuations: 24 in the placebo arm, 17 in the tiotropium arm. Similar to results for cardiac arrhythmia, Kaplan-Meier analysis of patients with an MI revealed a trend to later onset of the first MI event in the tiotropium arm compared with the placebo arm (Figure 1B).

Mean treatment duration following the first MI event was 581.7 days (SD: 444.1) in the placebo arm and 580.6 days (SD: 427.6) in the tiotropium arm. Among those patients administered placebo who had experienced an MI during the study, 55.9% subsequently experienced an SAE and 27.9% experienced a cardiac SAE. In the tiotropium arm, 68.3% of patients subsequently experienced an SAE and 28.6% experienced a cardiac SAE (Table 1).

Vital status FAEs in patients who had experienced an MI occurred with similar frequency in both those who had received placebo (16.2%) or tiotropium (12.7%) (Table 1). On-treatment FAEs were experienced by 16.2% of patients in the placebo arm and by 9.5% of patients in the tiotropium arm (Table 1).

Evaluation of the events on treatment by MACE endpoints did not suggest an increase in events with tiotropium compared with placebo for any of the measures (Table 1; Additional file 2). For patients who had an MI, the incidence of MACE was 16.2% in the placebo arm compared with 9.5% in the tiotropium arm. Fatal MACE (and fatal MACE including death unknown) affected 7.4% of patients receiving placebo and 4.8% of patients treated with tiotropium.

### Cardiac and mortality outcomes for patients with cardiac failure during the UPLIFT® study

During the study period, 397 patients experienced cardiac failure: 213 within the placebo arm, 184 in the tiotropium arm. Following the first event, there were 56 discontinuations: 27 in the placebo arm, 29 in the tiotropium arm. Kaplan-Meier analysis revealed a trend to later onset of the first cardiac failure event with tiotropium compared with placebo (Figure 1C).

Mean treatment duration following the first cardiac failure was 474.3 days (SD: 417.6) in the placebo arm and 447.0 days (SD: 399.4) in the tiotropium arm. Among those administered placebo who experienced cardiac failure during the study, 60.8% subsequently experienced an SAE and 23.7% experienced a cardiac SAE. In the tiotropium arm, 58.7% of patients subsequently experienced an SAE and 28.4% experienced a cardiac SAE (Table 1).

Following a cardiac failure event, vital status FAEs occurred with similar frequency in both those who had received placebo (22.0%) and those on tiotropium (21.9%) (Table 1). On-treatment FAEs were experienced by 16.7% of patients in the placebo arm and 19.4% of patients in the tiotropium arm (Table 1).

Evaluation of events on treatment by MACE endpoints did not suggest an increase in events with tiotropium compared with placebo (Table 1; Additional file 2). For patients experiencing cardiac failure, the incidence of MACE was 12.4% in the placebo arm compared with 10.3% in the tiotropium arm. Fatal MACE affected 7.0% of patients receiving placebo and 7.1% of patients treated with tiotropium; the incidence of fatal MACE, including death unknown, was 8.6% and 7.1%, respectively.

## Discussion

The UPLIFT® study confirmed the CV safety of tiotropium over 4 years for the population included [10,21]. The extensive, long-term patient data generated from the UPLIFT® study have allowed us to investigate retrospectively the potential impact of tiotropium versus placebo on the safety of patients experiencing the types of cardiac events during the study that would have led to exclusion at baseline. The analysis of SAEs or FAEs, including cardiac deaths and death for unknown reasons, in patients after experiencing cardiac arrhythmia, MI or cardiac failure during the conduct of UPLIFT® does not indicate any increase in total or cardiac SAEs or mortality with tiotropium HandiHaler® treatment compared with placebo.

The findings should ameliorate the concerns raised by Walker and colleagues regarding the generalisability of the results of the UPLIFT® trial, based on their analysis of a hospitalised population of patients with COPD in New Zealand [18]. Although patients with unstable or life-threatening cardiac arrhythmias, recent acute MI or severe heart failure requiring hospitalisation were excluded from UPLIFT® (in line with most long-term COPD trials designed to evaluate chronic benefit to risk of pharmacotherapy), but they were not required to withdraw from the study if such an event occurred. The majority of these patients remained in the trial, even though approximately 60% had an SAE and 20% had a cardiac SAE, with similar incidences in the tiotropium and placebo arms. Through the analysis of outcomes in these patients, we are able to show that tiotropium does not increase the risk of total or cardiac SAEs or mortality compared with placebo in patients who have previously experienced serious cardiac events.

It should also be noted that the hospitalised population described by Walker and colleagues [18] – in which 38% would have been ineligible for UPLIFT® at the time of discharge due to recent CV co-morbidity or moderate to severe renal impairment – may not be representative of the general COPD population. In a recent epidemiological analysis from the Netherlands (mean age 68 years), only 2.1% of patients starting treatment with tiotropium HandiHaler® had a COPD-related hospitalisation in the preceding year [22]. In addition, in an analysis of an elderly Canadian population (aged ≥66 years), only 9.8% were recently hospitalised for a respiratory condition and only 1.3% of patients were hospitalised for acute coronary syndrome, including MI, during the 6 months preceding the analysis [23]. Respective percentages were 0.2% for arrhythmias and 2.2% for heart failure. These findings suggest that the incidence of the conditions excluding patients from the initial inclusion in the UPLIFT® study is relatively low.

This analysis has strengths and weaknesses. One obvious weakness is that it is a *post-hoc* analysis with no hypothesis testing. However, the data from this analysis do not suggest a safety issue in patients with the respective cardiac conditions. Furthermore, a minority of patients dropped out due to the first cardiac event. In general, there was a trend towards fewer patients dropping out in the tiotropium group, limiting potential bias in favour of tiotropium. Strengths of the analysis are that it is derived from a randomised, double-blind study, which reduces the probability of bias in epidemiological analyses where effects arising from prognostic differences among patient groups are a major concern. Finally, the long, 4-year duration of UPLIFT® offered a unique opportunity to investigate the safety of tiotropium in patients with acute major cardiac events who remained in the study.

From this *post-hoc* analysis of the UPLIFT® trial data, we conclude that tiotropium HandiHaler® does not increase the risk of cardiac deaths, deaths unknown or other cardiac SAEs, following the occurrence of a cardiac event. The findings add to the body of data supporting the use of tiotropium HandiHaler® in patients with COPD, irrespective of pre-existing cardiac conditions.

## Additional files

Additional file 1:
**List of Institutional Review Boards (IRBs), Independent Ethics Committees (IECs) and consent forms for the UPLIFT® trial.**
Additional file 2:
**Breakdown of fatal MACE, fatal MACE (including death unknown) and MACE endpoints in patients with cardiac AEs during UPLIFT** **®** **(on treatment + 30 days)*.**

## Abbreviations

AE: Adverse event
CI: Confidence interval
COPD: Chronic obstructive pulmonary disease
CV: Cardiovascular
FAE: Fatal adverse event
MACE: Major adverse cardiovascular event
MedDRA: Medical Dictionary for Regulatory Activities
MI: Myocardial infarction
SAE: Serious adverse event
SD: Standard deviation
SMQ: Standardised Medical Dictionary for Regulatory Activities Query
SOC: System organ class
TIOSPIR™: TIOtropium Safety and Performance In Respimat®
UPLIFT®: Understanding Potential Long-term Impacts on Function with Tiotropium

## References

[1] Summary of Product Characteristics (SPC): Spiriva Respimat 2.5 microgram, inhalation solution [http://www.medicines.org.uk/emc/medicine/20134/SPC]

[2] Montuschi P, Macagno F, Valente S, Fuso L (2013). Inhaled muscarinic acetylcholine receptor antagonists for treatment of COPD. Curr Med Chem.

[3] Montuschi P, Ciabattoni G. Bronchodilating drugs for chronic obstructive pulmonary disease: current status and future trends. J Med Chem. 2015;58:4131–64. 10.1021/jm5013227 25587755

[4] Hochrainer D, Holz H, Kreher C, Scaffidi L, Spallek M, Wachtel H (2005). Comparison of the aerosol velocity and spray duration of Respimat Soft Mist inhaler and pressurized metered dose inhalers. J Aerosol Med.

[5] Pitcairn G, Reader S, Pavia D, Newman S (2005). Deposition of corticosteroid aerosol in the human lung by Respimat Soft Mist inhaler compared to deposition by metered dose inhaler or by Turbuhaler dry powder inhaler. J Aerosol Med.

[6] Ichinose M, Fujimoto T, Fukuchi Y (2010). Tiotropium 5microg via Respimat and 18microg via HandiHaler; efficacy and safety in Japanese COPD patients. Respir Med.

[7] van Noord JA, Cornelissen PJ, Aumann JL, Platz J, Mueller A, Fogarty C (2009). The efficacy of tiotropium administered via Respimat Soft Mist Inhaler or HandiHaler in COPD patients. Respir Med.

[8] Wise RA, Anzueto A, Calverley P, Dahl R, Dusser D, Pledger G (2013). The Tiotropium Safety and Performance in Respimat Trial (TIOSPIR), a large scale, randomized, controlled, parallel-group trial-design and rationale. Respir Res.

[9] Tashkin DP, Celli B, Senn S, Burkhart D, Kesten S, Menjoge S (2008). A 4-year trial of tiotropium in chronic obstructive pulmonary disease. N Engl J Med.

[10] Barr RG, Bourbeau J, Camargo CA, Ram FS (2006). Tiotropium for stable chronic obstructive pulmonary disease: a meta-analysis. Thorax.

[11] Kesten S, Leimer I, Jara M (2009). Risk of major adverse cardiovascular events with inhaled anticholinergics in patients with chronic obstructive pulmonary disease. JAMA.

[12] Rodrigo GJ, Castro-Rodriguez JA, Nannini LJ, Plaza MV, Schiavi EA (2009). Tiotropium and risk for fatal and nonfatal cardiovascular events in patients with chronic obstructive pulmonary disease: systematic review with meta-analysis. Respir Med.

[13] Salpeter SR (2009). Do inhaled anticholinergics increase or decrease the risk of major cardiovascular events?: a synthesis of the available evidence. Drugs.

[14] Tiotropium (Spiriva) Respimat: evaluation of fatal events [http://trials.boehringer-ingelheim.com/content/dam/internet/opu/clinicaltrial/com_EN/results/Pooled%20analysis/PA_205.372_251_252_254_255_U10-3255-01.pdf].

[15] Dong YH, Lin HH, Shau WY, Wu YC, Chang CH, Lai MS (2013). Comparative safety of inhaled medications in patients with chronic obstructive pulmonary disease: systematic review and mixed treatment comparison meta-analysis of randomised controlled trials. Thorax.

[16] Karner C, Chong J, Poole P (2012). Tiotropium versus placebo for chronic obstructive pulmonary disease. Cochrane Database Syst Rev.

[17] Singh S, Loke YK, Enright PL, Furberg CD (2011). Mortality associated with tiotropium mist inhaler in patients with chronic obstructive pulmonary disease: systematic review and meta-analysis of randomised controlled trials. BMJ.

[18] Walker S, Fingleton J, Weatherall M, Beasley R (2013). Limited generalisability of UPLIFT findings to clinical practice. Thorax.

[19] Tashkin DP, Metzdorf N, Hallmann C, Leimer I, Lee T, Decramer M (2014). Authors’ response to Walker et al. Thorax.

[20] Loke YK, Singh S, Furberg CD (2014). Tiotropium and the risk of death in COPD. N Engl J Med.

[21] Celli B, Decramer M, Kesten S, Liu D, Mehra S, Tashkin DP (2009). Mortality in the 4-year trial of tiotropium (UPLIFT) in patients with chronic obstructive pulmonary disease. Am J Respir Crit Care Med.

[22] Verhamme KM, Afonso A, Romio S, Stricker BC, Brusselle G, Sturkenboom M (2013). Use of tiotropium Respimat® SMI vs. tiotropium Handihaler® and mortality in patients with COPD. Eur Respir J.

[23] Gershon A, Croxford R, Calzavara A, To T, Stanbrook MB, Upshur R (2013). Cardiovascular safety of inhaled long-acting bronchodilators in individuals with chronic obstructive pulmonary disease. JAMA Intern Med.

