# Supplementary material for: Cardiac safety of tiotropium in patients with cardiac events: a retrospective analysis of the UPLIFT® trial
Source: Respir Res. 2015 Jun 2;16(1):65. doi: 10.1186/s12931-015-0216-4 (PMC4475325; doi:10.1186/s12931-015-0216-4)
Supplement: Additional file 2: — Breakdown of fatal MACE, fatal MACE (including death unknown) and MACE endpoints in patients with cardiac AEs during UPLIFT ® (on treatment + 30 days)*. [file 12931_2015_216_MOESM2_ESM.docx]

**Additional file 2: Breakdown of fatal MACE, fatal MACE (including death unknown) and MACE endpoints in patients with cardiac AEs during UPLIFT^®^ (on treatment + 30 days)***

|  | **Patients with event, n (%)** | | | | | |
| --- | --- | --- | --- | --- | --- | --- |
| **First event** | **Cardiac arrhythmia**^†^ | | **MI**^†^ | | **Cardiac failure**^†^ | |
| **Treatment arm/**  **Subsequent event** | **Placebo**  **(n = 181)** | **Tiotropium HandiHaler^®^  18 μg**  **(n = 193)** | **Placebo**  **(n = 68)** | **Tiotropium HandiHaler^®^  18 μg**  **(n = 63)** | **Placebo**  **(n = 186)** | **Tiotropium HandiHaler^®^ 18 μg**  **(n = 155)** |
| Fatal MACE | 15 (8.3) | 10 (5.2) | 5 (7.4) | 3 (4.8) | 13 (7.0) | 11 (7.1) |
| Cardiac disorders SOC (fatal) | 13 (7.2) | 9 (4.7) | 3 (4.4) | 3 (4.8) | 11 (5.9) | 9 (5.8) |
| SMQ ischaemic heart disease sub-SMQ  MI (broad) (fatal) | 4 (2.2) | 1 (0.5) | 2 (2.9) | 0 (0.0) | 3 (1.6) | 1 (0.6) |
| Stroke (fatal) | 1 (0.6) | 1 (0.5) | 1 (1.5) | 0 (0.0) | 1 (0.5) | 0 (0.0) |
| Sudden death PT | 1 (0.6) | 0 (0.0) | 1 (1.5) | 0 (0.0) | 0 (0.0) | 2 (1.3) |
| Vascular disorders SOC (fatal) | - | - | - | - | 1 (0.5) | 0 (0.0) |
| Fatal MACE (including death unknown) | 18 (9.9) | 13 (6.7) | 5 (7.4) | 3 (4.8) | 16 (8.6) | 11 (7.1) |
| Cardiac disorders SOC (fatal) | 13 (7.2) | 9 (4.7) | 3 (4.4) | 3 (4.8) | 11 (5.9) | 9 (5.8) |
| Death PT | 3 (1.7) | 3 (1.6) | - | - | 3 (1.6) | 0 (0.0) |
| SMQ ischaemic heart disease sub-SMQ MI (broad) (fatal) | 4 (2.2) | 1 (0.5) | 2 (2.9) | 0 (0.0) | 3 (1.6) | 1 (0.6) |
| Stroke (fatal) | 1 (0.6) | 1 (0.5) | 1 (1.5) | 0 (0.0) | 1 (0.5) | 0 (0.0) |
| Sudden death PT | 1 (0.6) | 0 (0.0) | 1 (1.5) | 0 (0.0) | 0 (0.0) | 2 (1.3) |
| Vascular disorders SOC (fatal) | - | - | - | - | 1 (0.5) | 0 (0.0) |
| MACE | 25 (13.8) | 15 (7.8) | 11 (16.2) | 6 (9.5) | 23 (12.4) | 16 (10.3) |
| Cardiac disorders SOC (fatal) | 13 (7.2) | 9 (4.7) | 3 (4.4) | 3 (4.8) | 11 (5.9) | 9 (5.8) |
| SMQ ischaemic heart disease sub-SMQ MI (broad) (any) | 6 (3.3) | 5 (2.6) | 7 (10.3) | 3 (4.8) | 9 (4.8) | 8 (5.2) |
| Stroke (any) | 10 (5.5) | 4 (2.1) | 3 (4.4) | 0 (0.0) | 6 (3.2) | 0 (0.0) |
| Sudden death PT | 1 (0.6) | 0 (0.0) | 1 (1.5) | 0 (0.0) | 0 (0.0) | 2 (1.3) |
| Vascular disorders SOC (fatal) | - | - | - | - | 1 (0.5) | 0 (0.0) |

*Not exposure corrected. ^†^First event did not lead to discontinuation.

AE, adverse event; MACE, major adverse cardiovascular event; MI, myocardial infarction; PT, preferred term; SMQ, Standardised Medical Dictionary for Regulatory Activities Query; SOC, system organ class; UPLIFT^®^, Understanding Potential Long-term Impacts on Function with Tiotropium.
